# Identifying factors linked to the occurrence of alien gastropods in isolated woodland water bodies

**DOI:** 10.1007/s00114-014-1153-7

**Published:** 2014-02-07

**Authors:** Aneta Spyra, Małgorzata Strzelec

**Affiliations:** Department of Hydrobiology, Faculty of Biology and Environmental Protection, The University of Silesia, 9 Bankowa Street, 40-007 Katowice, Poland

**Keywords:** Alien species, Freshwater gastropods, Spread, Colonisation, Woodland ponds, Anthropogenic reservoirs

## Abstract

Biological invasions are a significant component of human-caused global change and is widely regarded as one of the main threats to natural biodiversity. Isolated anthropogenic water bodies created in the areas that are deprived of natural freshwater habitats allow the survival and reproduction of alien species on newly settled sites. They are often small with water level fluctuations causing frequent environmental disturbances. The colonisation success may be the result of the rate of their degradation. The aims of the study were to determine the environmental conditions that affect the existence of alien species of gastropods in this type of aquatic environment and to examine whether the occurrence of non-native species affects the community structure of the native species. This study made it possible to group woodland ponds according to the occurrence of the three invasive species in snail communities and discuss the environmental conditions present in these pond types. Analysis of water properties emphasised the distinctiveness of the selected pond types. In ponds of the *Potamopyrgus antipodarum* type, we found the highest values of some parameters mainly hardness, conductivity, and content of calcium and chlorides, in contrast with the *Physella acuta* type, which were characterised by the lowest values except for phosphates and nitrites. In the *Ferrissia fragilis* type, we found the highest nitrate content. Data on the occurrence of alien species in different water environments play an important role in actions which are taken to prevent new invasions and spread of non-native species as well as to reduce future impacts of invaders.

## Introduction

The colonisation of new areas by alien gastropods is an important problem due to the actions that have been taken in recent years to protect valuable habitats that are important for preserving biological diversity. Anthropogenic woodland ponds of different origins, which are isolated from urban areas and which are formed as a new element in the landscape of forest areas, belong to this type of aquatic environment.

Alien gastropods increasingly appear in such environments. *Physella cauta* is commonly present around the Mediterranean Sea, while *Potamopyrgus antipodarum* originates from New Zealand and *Ferrissia fragilis* belongs to North American species (Walther et al. 2006).

There are different paths of the migration of gastropods into isolated reservoirs. The two main paths are a natural, slow and non-directional spreading and an anthropogenic expansion with the participation of human activity (Nentwig 2007). The isolation of woodland ponds and the relatively small distance between them mean that water birds may be involved in the dispersion process (Van Leeuwen et al. 2012a, b, c). This kind of passive dispersion has been described in the case of *P. antipodarum* (Haynes et al. 1985; Zaranko et al. 1997; Alonzo and Castro-Diez 2008), although anthropogenic dispersion (Devin et al. 2005) or accidental introductions should not be excluded.

The success of the colonisation of alien gastropods is widely regarded as one of the main threats to natural biodiversity (Kolar and Lodge 2001). This is possibly due to the specific features of their biology, which affect the rate of population growth. Such features include the time it takes to achieve sexual maturity (Tibbets et al. 2010), their high degree of fertility (Richards 2002), the type of reproduction (e.g. parthenogenesis in a colonised area—*P. antipodarum*) (Lively 1992), as well as their ability to tolerate a wide range of environmental conditions (e.g. a high tolerance to extreme temperatures in the case of *P. antipodarum* and *Physella acuta* (Jaeckel 1962; Winterboum 1969; Hylleberg and Siegismund 1987; Strzelec 1999; Van Leeuwen et al. 2013) and most importantly their huge evolution potential, which allows them to adapt to new environmental conditions (Hänfling and Kollmann 2002). The success of colonisation may also be the result of the low diversity of microhabitats in woodland ponds and the varying degrees of their degradation, although according to Pip (1986), the small surface area may be the reason for the reduction in the rate of migrational exchange (small populations) in comparison to large ponds.

According to Havel et al. (2005), water reservoirs offer new opportunities for colonisation as is exemplified by the increasing number of sites with populations of *P. antipodarum*, *F. fragilis* and *P. acuta* that have been recorded more and more frequently in recent years in different countries and also in Poland (Meier-Brook 2002; Spyra 2008). There is a general agreement that it is more likely that alien species will be found in disturbed rather than in undisturbed environments (Früh et al. 2012a, b). Anthropogenic woodland ponds can be classified as the first type, since they are a product of human pressure and remain under its influence. Disturbances (e.g. drainage, a reduction in the level of groundwater and degradation as a result of acidification and the input of pollutants) create ‘open niches’ of invasion (Lockwood et al. 2007; Lodge 1993a, b).

The aims of this study were to answer two questions: (1) Is the occurrence of the three non-native species related to a particular environmental condition?; and (2) does the occurrence of non-native species affect the community structure of the native species?

## Material and methods

### Study sites

**Fig. 1:**
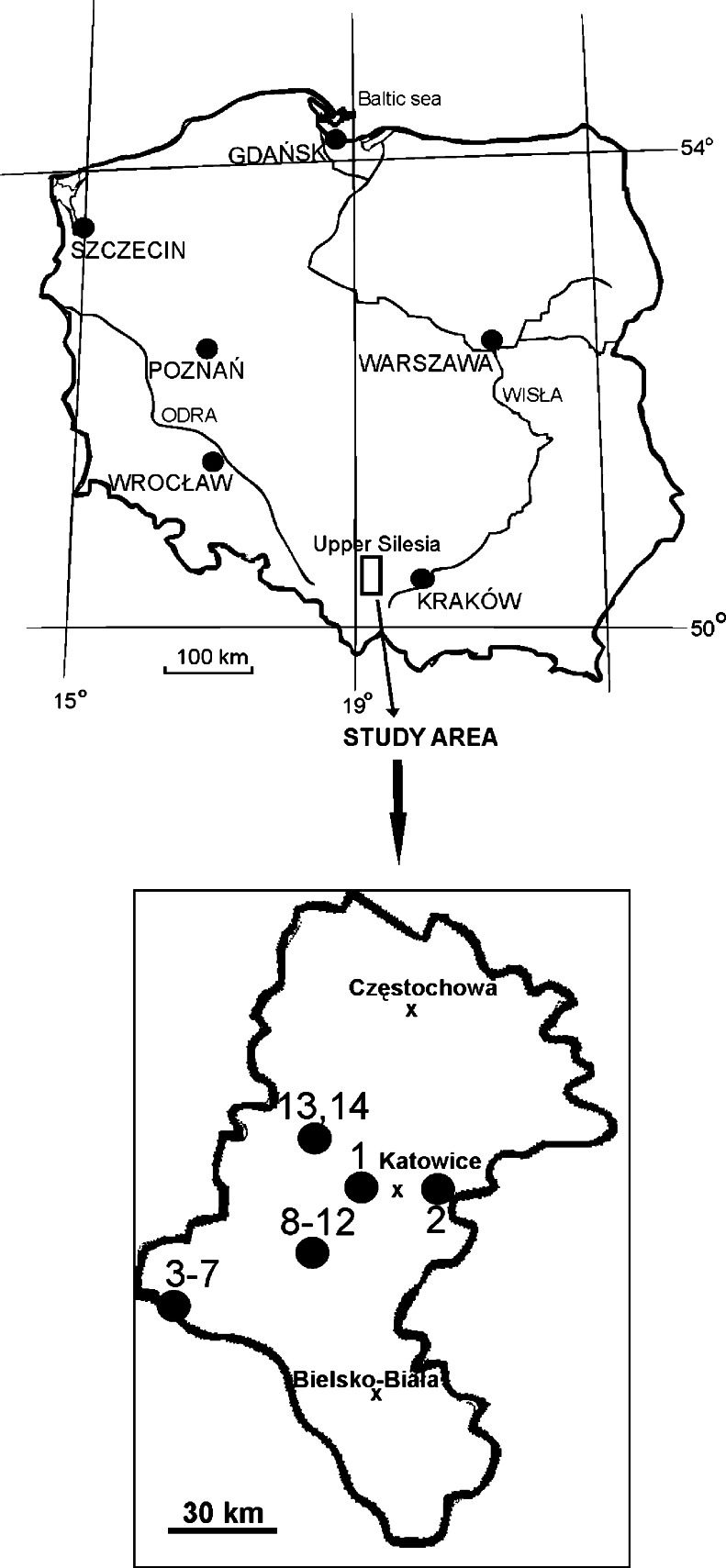
Location of the woodland water bodies studied in Southern Poland

The study was carried out in 14 isolated ponds located in forest complexes in southern Poland (Upper Silesia). The ponds are mainly post-exploitation: sand pits (ponds 2 and 13), gravel pits (ponds 3–7) and subsidence ponds (ponds 1, 8–12 and 14) (Table 1, Fig. 1). They are not used economically or recreationally. All of the ponds that were studied are ‘young’ (the oldest originated in 1945 and the youngest in the 1990s of the twentieth century), small (from 0.007 to 11.4 ha) and shallow (max. depth does not exceed 6 m).

**Table 1:** Environmental features of the three types of woodland ponds studied

Parameter	Type of water body		
	*P. acuta*	*F. fragilis*	*P. antipodarum*
Area (ha)	0.007–8.5	6.7–11.4	3.0–3.5
Maximal depth (m)	1–6	2–3.5	2–3.6
Number of water bodies	5	4	5
Number of study sites	14	10	8
Year of origin	1960–1980	1945–1990	1970–1993
Source of water supply	Surface run-off, ground water, rainfall	Surface run-off, ground water, rainfall, woodland drainage ditches	Surface run-off, ground water, rainfall, woodland drainage ditches

They are similar to natural, small, eutrophic ponds, but their origin is the reason for the specific physico-chemical properties of the water. Because of the way water is supplied, they are characterised by significant fluctuations in water levels. This situation is best illustrated by the water withdrawal that exposes detritus deposits of various origins. High water levels occur during the spring after the snow melts as well as after intensive rainfalls. In the winter, ponds freeze partially or completely to the bottom in the shore area. Because of continued land subsidence, some parts of the shores have been reinforced by concrete slabs. This prevents the adjacent areas from becoming wet. As time passes, the surfaces of the concrete slabs have become sites where aquatic vegetation has grown as well as places for the accumulation of detritus and algae. The riparian zone of the ponds is composed of deciduous trees (mainly *Quercus robur*, *Quercus rubra*, *Populus tremula*, *Betula pendula*, *Alnus glutinosa*).

### Site selection

Due to the possibility of the colonisation of various substrates by gastropods, the study sites were selected taking into account all of the commonly occurring types:

1. Macrophytes (11 sampling sites)—samples were collected in the rushes from both living plants and their remains.
2. Leaf deposits (11 sampling sites)—the most common type of substrate in woodland ponds that cover the surface of sediments depending on the origin of the water body—mud, gravel or sand.
3. Anthropogenic substrates (10 sampling sites)—samples were taken from the concrete slabs with a layer of algae and detritus that reinforced the shores that were submerged in the water.

Sampling sites were located along the shoreline of the ponds. The water depth at the place of sampling did not exceed 0.5 m. Thirty-two study sites were selected in 14 ponds.

### Data collection and analysis

The samples were taken in 2003 (ponds 1, 2, 3, 4, 5, 6, 7), 2008 (ponds 8, 9, 10, 11, 12, 13,) and 2010 (pond 14). Samples were collected once a month from April to October at each of the selected study sites. During July, six study sites were dry, thus making sampling impossible. In total, 218 samples that were dug to a depth of 0.2 m were collected using a square frame (0.5 × 0.5 m). We use a smaller mesh sieve diameter than is usually used (0.5 mm) in order to take into account all gastropods and juvenile specimens. Gastropods were preserved in 75 % ethanol. They were identified according to Glöer (2002) based on the morphological features of the shell with the exception of *Radix auricularia* and *Radix balthica*. For their determination, we also included pigmentation and, in taxonomically doubtful cases, the anatomical reproductive features according to Jackiewicz (2000). Juvenile stages were identified according to Glöer (2002), and in doubtful cases, these specimens were bred in aquariums and their identification was verified and confirmed after a period of their growth. The density of gastropods was estimated as the number of individuals per 1 m^2^.

Prior to the sampling of gastropods, water samples were collected from each sample site. Analyses of some of the physical and chemical parameters of water that might affect the occurrence of gastropods were carried out using standard methods, according to Hermanowicz et al. (1999). Only the water temperature was measured in the field (Table 2).

**Table 2:** The ranges and means ( $\overline{x}$) of the physical and chemical parameters of water in the three types of woodland ponds studied

Parameter	Type of water body					
	*P. acuta*		*F. fragilis*		*P. antipodarum*	
pH	6.6–9.5	$\overline{x}$= 7.8	5.9–7.4	$\overline{x}$= 7.1	4.0–9.0	$\overline{x}$= 6.0
Total hardness (dH)	1.4–9.0	$\overline{x}$= 6.1	2.1–48	$\overline{x}$= 12.1	6.8–138	$\overline{x}$= 51.1
Conductivity (μS cm)	120–150	$\overline{x}$= 299	60–890	$\overline{x}$= 464	230–6,860	$\overline{x}$= 3,048
Calcium (mg dm^−3^)	13–68	$\overline{x}$= 36.2	13–123	$\overline{x}$= 69	29–314	$\overline{x}$= 101
Chlorides (mg dm^−3^)	10–60	$\overline{x}$= 26	5–79	$\overline{x}$= 45	25–335	$\overline{x}$= 199
N-NH_3_ (mg dm^−3^)	0–0.9	$\overline{x}$= 0.3	0.02–0.82	$\overline{x}$= 0.5	0.04–8.2	$\overline{x}$= 4.1
Nitrite (mg NO_2_ dm^−3^)	0–0.2	$\overline{x}$= 0.18	0.002–0.16	$\overline{x}$= 0.02	0–0.15	$\overline{x}$= 0.03
Nitrates (mg NO_3_ dm^−3^)	0–12.85	$\overline{x}$= 3.68	0–29.2	$\overline{x}$= 11.6	0–11.5	$\overline{x}$= 3.4
Phosphate (mg PO_4_ dm^−3^)	0.01–0.89	$\overline{x}$= 0.69	0–0.62	$\overline{x}$= 0.31	0.02–0.52	$\overline{x}$= 0.23
Total dissolved solids (mg dm^−3^)	50–250	$\overline{x}$= 147	30–430	$\overline{x}$= 244	120–2,250	$\overline{x}$= 1,030

The gastropod community analysis was carried out using the following indices:

1. Dominance index (*D*) in percent of the total number of individuals in the whole collection: *D* = *k* / *K* × 100, where *k* is the number of individuals of species and *K* is the total number of individuals in a sample. The following dominance classes were used (Biesiadka and Kowalik 1980): *D* > 10 %—eudominants, *D* = 5.1–10 %—dominants, *D* = 2.0–5.0 %—subdominants and *D* < 2.0 %—recedents.
2. Simpson’s diversity index: expressed as $S=1-{\displaystyle \sum_{i=1}^S}\overset{2}{\left(\mathrm{pi}\right)}$, where pi is the relative abundance of the *i* species in a community.
3. The evenness index: *J*′ = *H*′ / log_2_ *S*, where *H*′ is the value of the Shannon-Wiener index and *S* the total number of species.

The species diversity index as well as the dominance index was calculated for each of the sampling sites and for the specific ponds.

### Statistical analysis

The results were analysed with STATISTICA ver. 11.0. The cluster analysis was used to distinguish the three types of woodland ponds concerning their faunistic similarities. The dendrogram was based on the abundance of a particular snail species. Euclidean distances were used in the tree clustering method.

We used the Spearman rank correlation coefficients (*r*_s_) to analyse the relationships between the density of alien species, species richness and the age of the ponds, as well as the proportion of alien gastropods in a community (each of the three species separately and all of the species combined) versus the species diversity, species richness, species evenness and density.

We used the Kruskal-Wallis one-way ANOVA and multiple comparisons (post hoc tests) to determine the significance of the differences between the environmental conditions (age, area and chemical water parameters) of the three pond types. Before analysis, the data distribution was checked using the Kolmogorov-Smirnov and Lilliefors tests (Stanisz 2001). These demonstrated that the variables did not show a normal distribution (K-S *p* < 0.20 and Lilliefors *p* < 0.20). For this reason, non-parametric statistics were used (Elliott 1993).

A canonical correspondence analysis (CCA) was carried out using CANOCO 4.5 software (Ter Braak 2002) in order to elucidate the relationships between the composition of the gastropod communities and the environmental parameters. The appropriate type of analysis was chosen to analyse the species data using detrended correspondence analysis (DCA) and the length of the gradient. Preliminary DCA on the species data revealed that the gradient length was more than 3 SD (4.6 SD), thus indicating that the species exhibited unimodal responses to underlying environmental variations, which justified the use of the unimodal, direct type of analysis. Therefore, a unimodal, direct ordination CCA with a forward selection was used. The significance of the relationships between the gastropod species ordination and environmental variables, as well as the axes, was tested in the forward selection procedure using the Monte Carlo permutation test. Prior to analysis, environmental data were log-transformed ln(*x* + 1) (Ter Braak 2002). CCA analysis was performed using a selection in which the Monte Carlo test of significance for all variables was assessed and only then were statistically significant variables taken into account for further analysis. An ordination diagram based on variables that statistically significantly influenced the occurrence of gastropods was made using the program CanoDraw.

In the CCA analysis, the following environmental variables were used: chemical water properties, different types of substrates (macrophytes, leaf deposits and anthropogenic substrates), pond depth, pond size, maximum water temperature, pond freezing in the winter and pond drying in the summer. These variables can significantly affect the occurrence of alien and native gastropods. The physico-chemical properties of water in woodland ponds are specific due to their isolation, fluctuations in the water level and drying. The structure of the substrates also changes as a result of drying and freezing.

## Results

**Fig. 2:**
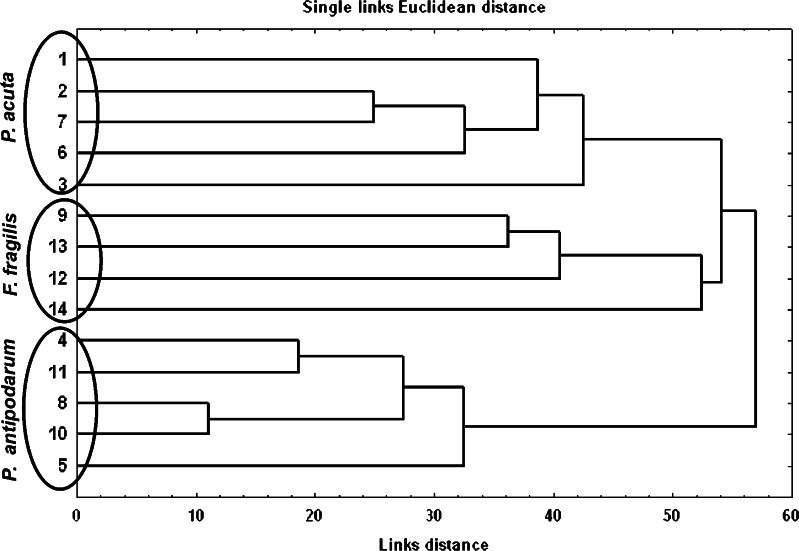
Dendrogram of the faunistic similarities of freshwater snails in the woodland ponds studied

Twenty-three gastropod species were detected in Polish fauna including three alien species. Only *Radix balthica* (O.F. Müller, 1774) and *Gyraulus alb*us (Müller, 1774) occurred most frequently. *Viviparus contectus* (Millet, 1813), *Anisus vortex* (Linnaeus, 1758), *Physa fontinalis* (Linnaeus, 1758) and *Aplexa hypnorum* (Linnaeus, 1758) were found in only a few ponds (Table 3). Cluster analysis provided the basis for distinguishing the three types of ponds (Fig. 2):

**Table 3:** Occurrence of freshwater snails in the woodland ponds studied

Species	Ponds													
	1	2	3	4	5	6	7	8	9	10	11	12	13	14
*Viviparus contectus*					✓									
*Potamopyrgus antipodarum*			✓	✓	✓		✓	✓		✓	✓			
*Bithynia tentaculata*		✓	✓	✓	✓									
*Valvata cristata*	✓				✓						✓			
*Valvata piscinalis*		✓	✓	✓		✓								
*Lymnaea stagnalis*		✓	✓	✓	✓							✓	✓	✓
*Stagnicola palustris*		✓						✓		✓			✓	
*Stagnicola corvus*			✓											✓
*Galba truncatula*	✓													✓
*Radix auricularia*		✓	✓	✓	✓								✓	
*Radix balthica*	✓	✓	✓	✓	✓	✓	✓	✓	✓	✓	✓	✓		✓
*Planorbis planorbis*	✓	✓		✓	✓		✓							✓
*Anisus spirorbis*					✓				✓	✓				
*Anisus vortex*														✓
*Bathyomphalus contortus*												✓	✓	
*Gyraulus albus*	✓	✓	✓	✓	✓	✓	✓	✓	✓	✓		✓	✓	✓
*Gyraulus crista*	✓	✓			✓					✓		✓		✓
*Hippeutis complanatus*	✓	✓							✓			✓	✓	✓
*Planorbarius corneus*	✓	✓							✓	✓		✓	✓	✓
*Ferrissia fragilis*	✓								✓			✓	✓	✓
*Physa fontinalis*												✓		
*Physella acuta*	✓	✓	✓	✓	✓	✓	✓							
*Aplexa hypnorum*										✓				
Number of species	10	12	9	9	12	4	5	4	6	8	3	13	8	11

1. *P. acuta* type (ponds 1, 2, 3, 6 and 7)—where *P. acuta* occurred constantly and other alien species were found in only a few ponds.
2. *F. fragilis* type (ponds 9, 12, 13 and 14)—where *F. fragilis* is the single alien species.
3. *P. antipodarum* type (ponds 4, 5, 8, 10 and 11)—where, in addition to *P. antipodarum*, *P. acuta* occurred sporadically in samples collected in ponds 4 and 5.

The chronology of their creation indicates that the *P. antipodarum* type is the youngest and the smallest type among forest ponds (Table 1). The differences in the age and area of the three distinguished pond types were statistically significant (Kruskal-Wallis: *F* = 28.41, *p* < 0.0001 and *F* = 22.21, *p* < 0.0001, respectively). The results of our study show that species richness and diversity do not depend on the age of the ponds (*r*_s_ = −0.13, *r*_s_ = −0.30, *p* > 0.05, respectively).

Analysis of physico-chemical water properties emphasised the distinctiveness of the selected types. In water bodies of the *P. antipodarum* type, we found the highest values of some parameters (Kruskal-Wallis: hardness *F* = 28.96, *p* = 0.01; conductivity *F* = 30.44, *p* = 0.005; total dissolved solids *F* = 19.22, *p* = 0.0009; Ca *F* = 23.9, *p* = 0.0001; Cl *F* = 27.86, *p* = 0.001; N-NH_3_ *F* = 14.86, *p* = 0.0001), while in the *P. acuta* type, we found their lowest values in comparison to the other types. The pH values in ponds of *P. antipodarum* type were lower in comparison to the other types (*F* = 28.24, *p* = 0.002). In ponds of the *P. acuta* type, we observed the highest content of phosphate and nitrite (*F* = 33.38, *p* = 0.001 and *F* = 13.82, *p* = 0.001, respectively), and in the *F. fragilis* type, the highest nitrate content (Kruskal-Wallis: 21.78, *p* = 0.001) (Table 2).

Water bodies of the *F. fragilis* type were characterised by the highest density of gastropods in contrast to the *P. acuta* type. Alien species were least numerous in water bodies of the *P. acuta* type (from 50 to 250 ind./m^2^) and most numerous in the *P. antipodarum* type (from 117 to 948 ind./m^2^). The alien species of a specific type did not reach high densities in the different pond types (Table 4). In ponds with a higher density of *P. antipodarum*, the density of *P. acuta* was low and vice versa (*r*_s_ = −0.56, *p* < 0.05).

**Table 4:** Characteristics of snail communities in the three types of woodland ponds studied

Characteristics of snail communities	Type of water body					
	*P. acuta*		*F. fragilis*		*P. antipodarum*	
Number of species in type of ponds	17		16		16	
Number of species in woodland ponds	4–12	$\overline{X}\kern0.5em =\kern0.5em 8$	6–13	$\overline{X}\kern0.5em =\kern0.5em 8$	3–12	$\overline{X}\kern0.5em =\kern0.5em 7$
Density of snails (ind./m^2^)	79–388	$\overline{X}\kern0.5em =\kern0.5em 182$	74–1,002	$\overline{X}\kern0.5em =\kern0.5em 548$	117–948	$\overline{X}\kern0.5em =\kern0.5em 429$
Density of an alien species (ind./m^2^)	7–97	$\overline{X}\kern0.5em =\kern0.5em 32$	3–202	$\overline{X}\kern0.5em =\kern0.5em 72$	3–536	$\overline{X}\kern0.5em =\kern0.5em 196$
Other alien species	*P. antipodarum* *F. fragilis*				*P. acuta*	

**Fig. 3:**
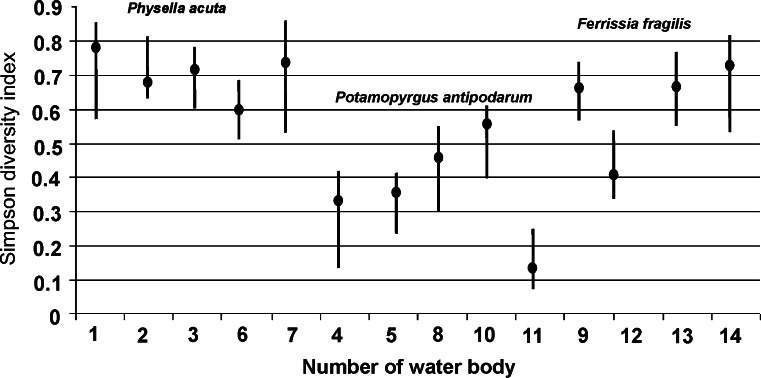
The value of the Simpson diversity index in the three types of woodland ponds. *Filled circle*, the value of Simpson diversity index calculated for woodland ponds in the study period; *vertical line*, the min and max value of Simpson diversity index calculated for the sampling sites in particular ponds

In each type of pond in addition to the dominant species, different gastropods belonged to eudominants. The number of subrecedents in each type emphasised their dominance patterns. In the ponds of the *P. antipodarum* type, 11 of the 16 reported species belonged to recedents (Table 5). This is also expressed in the values of the Simpson index (Fig. 3). Its highest value is characterised for gastropod communities in the ponds of the *P. acuta* type due to the number of eudominant and dominant species (3 and 5, respectively) and the participation of subdominants and recedents in the communities (3 and 6, respectively). Only the Simpson’s index value in pond 12 is similar to the values characterising the *P. antipodarum* type, which is the result of the high percentages of *Hippeutis complanatus* (Linnaeus, 1758) (Table 5, Fig. 3).

**Table 5:** Domination patterns [percent] in the three types of woodland ponds studied

Dominance classes	*P. acuta* type	*F. fragilis* type	*P. antipodarum* type
Eudominants	*B. tentaculata* 14.8	*G. crista* 20,1	*P. antipodarum* 26.8
	*V. piscinalis* 18,6	*H. complanatus* 37.6	*R. balthica* 52.1
	*P. acuta* 20,0	*F. fragilis* 12.7	
Dominants	*R. balthica* 8.2		
	*P. planorbis* 6.3	*R. auricularia* 6.6	*B. tentaculata* 6.4
	*G. albus* 5.7	*P. planorbis* 6.9	*G. albus* 7.7
	*H. complanatus* 8.9	*P. corneus* 5.1	
	*F. fragilis* 7.3		
Subdominants	*R. auricularia* 2.7	*L. stagnalis* 2.6	
	*G. crista* 2.5	*B. contortus* 2.1	*G. crista* 2.2
	*P. corneus* 3.4	*G. albus* 3.8	
Recedents			*V. contectus* 0.1
			*V. cristata* 0.5
		*S. palustris* 0.1	*V. piscinalis* 0.2
	*P. antipodarum* 0.7	*L. corvus* 0.1	*L. stagnalis* 0.1
	*V. cristata* 0.1	*G. truncatula* 0.1	*S. palustris* 0.2
	*L. stagnalis* 0.5	*R. balthica* 1.4	*R. auricularia* 0.5
	*S. palustris* 0.1	*A. spirorbis* 0.1	*P. planorbis* 0.7
	*L. corvus* 0.1	*A. vortex* 0.6	*A. spirorbis* 0.6
	*G. truncatula* 0.1	*P. fontinalis* 0.1	*P. corneus* 0.6
			*P. acuta* 1.2
			*A. hypnorum* 0,1

An analysis of Spearman rank correlation coefficients showed a significant correlation between the proportion of *P. antipodarum* in a community and species diversity (*r*_s_ = −0.56, *p* < 0.05). Correlations between alien species of gastropods and species diversity, species richness, species evenness and snail density were statistically insignificant.

**Fig. 4:**
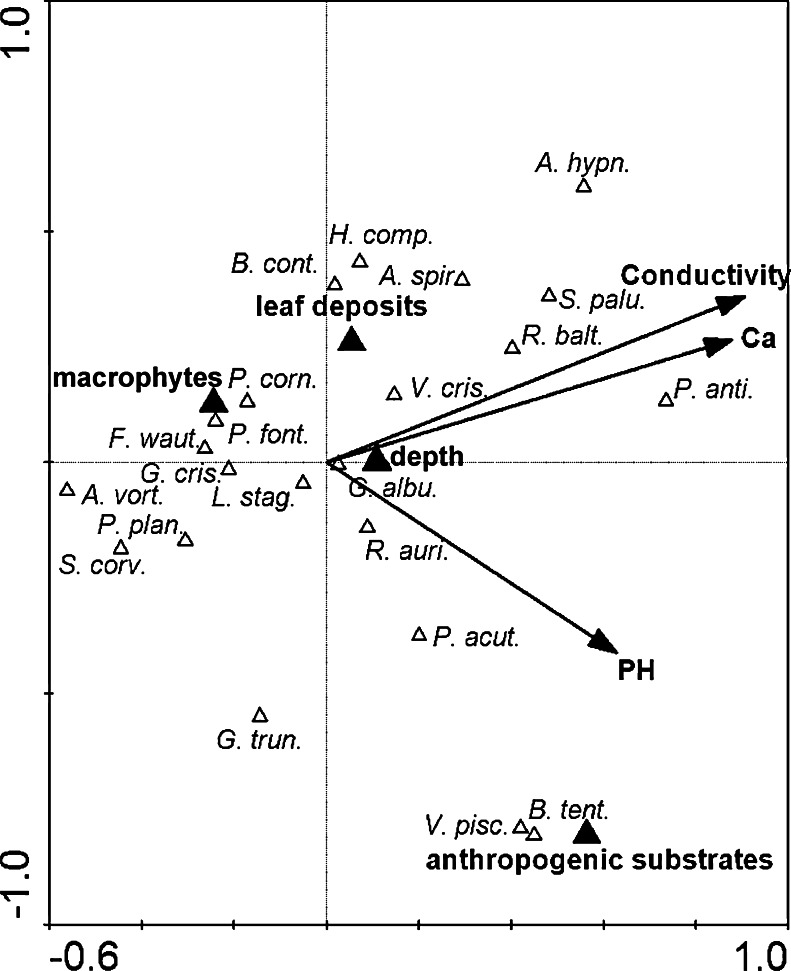
Canonical correspondence analysis (CCA) diagram showing the snail communities in relation to environmental parameters

**Fig. 5:**
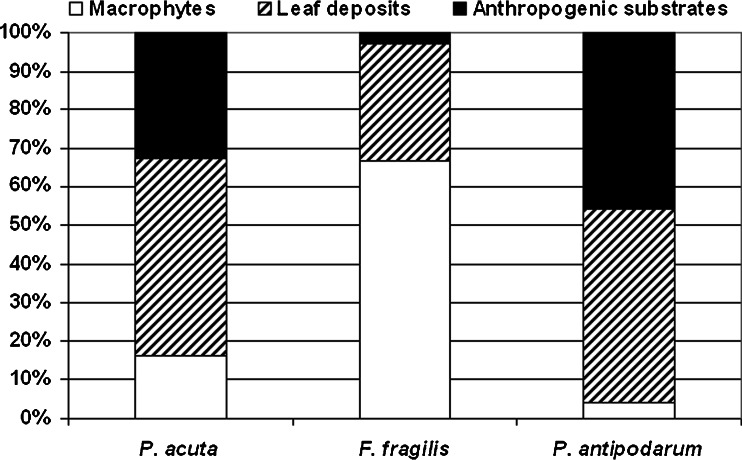
Occurrence of non-native species of snails in relation to different types of substratum

In the CCA analysis, gastropod communities showed high positive correlations with seven variables that reflected the variety of substrates, depth and differences in water chemistry (Fig. 4). The main variables that influenced the gastropod community were conductivity, Ca and pH. *F. fragilis* along with *Planorbarius corneus*, *Gyraulus crista* and *A. vortex* was mainly associated with macrophytes. Three species were associated with leaf deposits, whereas two species were related to anthropogenic substrates. *P. antipodarum* occurred in water with a high content of calcium and conductivity (Fig. 4). It occurred on all of the available types of substratum, but mostly in leaf deposits and anthropogenic substrates (Fig. 5). The depth of a water body mainly influenced the occurrence of *G. albus*. CCA showed that the first and second axes explain almost 18.6 % of the variance in species data and almost 62 % of the variance in species and environmental relations. The Monte Carlo test showed that these results are statistically significant (Table 6).

**Table 6:** Summary of the canonical correspondence analysis (CCA) carried out on freshwater snail species and environmental data

Axes	1	2	3	4	Total inertia
Eigenvalues	0.454	0.278	0.255	0.153	3.943
Species-environment correlations	0.889	0.778	0.866	0.705	
Cumulative percentage variance					
Of species data	11.5	18.6	25.0	28.9	
Of species-environment relation	32.9	62.2	70.2	81.1	
Sum of all eigenvalues					3.943
Sum of all canonical eigenvalues					1.406
Summary of the Monte Carlo test					
Test of the significance of first canonical axis: eigenvalues = 0.806					
*F*-ratio = 19.436					
*p* value = 0.0020					
Test of the significance of all canonical axes: trace = 2.839					
*F*-ratio = 7.959					
*p* value = 0.0020					

## Discussion

The area of southern Poland is heavily anthropogenically transformed due to the development of many branches of industry. There is a lack of natural water bodies, whereas those that exist were created as a result of intentional or unintentional human activity. For this reason, in our study, we did not include natural reservoirs. Anthropogenic woodland ponds create habitats for the occurrence of many gastropods. They constitute important trophic levels between primary producers and consumers and play a key role in shaping an aquatic biocenosis (Kerans et al. 2005).

The composition of gastropod assemblages is conditioned by the catchment area, and differences in the structure of their communities are most likely the result of the variability of many factors including the degree of the water shading, the organic matter, the physico-chemical water properties and substrate heterogeneity (Harman 1972; Økland 1990; Glöer and Diercking 2009). Woodland ponds are not always suitable habitats for gastropods; however, despite spatial isolation, they can be sites for the further spreading of alien species. Colonisation success is not attained by the specialised species that are typical of stable habitats, but rather by species with a tolerance to a wide range of environmental conditions, which was confirmed in our study. In the woodland ponds that were studied, we found the constant occurrence of *G. crista*, often found in highly eutrophic and polluted reservoirs (Beran 2002), and *R. balthica*, the first coloniser in newly created anthropogenic ponds (Reavel 1980), which is resistant to desiccation (Gérard 2001) and frequently occurs in waters with a high content of calcium and chloride (Dussart 1979).

Small water bodies, which originate from anthropogenic activity and remain under its influence, are characterised by a low diversity of gastropods (Pip 1986). According to Bronmark and Hansson (2000)), shallow (such as those in this study) and warm reservoirs with a high content of nutrients create a favourable habitat for the growth of algae, which are food for many herbivores such as *R. balthica* (Storey 1971). Woodland ponds create new habitats for flora and fauna, and the passive transport of individuals (being moved by animals, e.g. in the digestive system) has been recorded as being important for regional dispersion. Passive dispersal importantly contributes to biodiversity (Loson and MacIsaac 1997; Van Leeuwen et al. 2012a, b, c).

It is assumed that the alien species of gastropods that were found in the woodland ponds occurred without the participation of human activity breaking the barrier of the forest. At the time of their appearance in anthropogenic water bodies, they, as well as native species, face environmental stresses: hydrological, chemical and light, which are associated with the way their water is supplied, and which among other factors determine the properties of its waters.

The appearance of *P. antipodarum* and the other alien species in woodland ponds confirms their ability for dispersion using different paths of passive transport (according to the classification of Alonzo and Castro-Diez 2008) with regard to the location. Their colonisation success is also connected with the lack of competition from established native species in these ponds which are also a significant factor in the rate of colonisation. According to Schreiber et al. (2003), environmental disturbances associated with human activity can facilitate this species settling into new areas by creating spaces for colonisation. This species can potentially be found around the world at sites with similar ecological characteristics. Confirmation of this fact is demonstrated in our study. We found a lack of preference of *P. antipodarum* for a certain type of substrate as opposed to *F. fragilis* for which the presence of macrophytes constitutes the optimal habitat.

The three types of ponds that were distinguished differ in relation to the structure of the freshwater gastropods communities, the physico-chemical water properties and, in some cases, the age and area. The least stable community structure was found in the youngest ponds—the *P. antipodarum* type. This is confirmed by the number of recedents, eudominants and dominants. Many more species belong to the recedents than to other dominance classes. This is typical for habitats with significant degradation, e.g. with high values of conductivity or total dissolved solids such as those characterised by water bodies of the *P. antipodarum* type. The numerous occurrence of *R. balthica* in woodland water bodies of the above type confirms its preference for water with a high content of chlorides and calcium (Dussart 1977).

The most stable dominance structure was found in ponds of the *P. acuta* type, where 11 of the 17 recorded species of gastropods belonged to eudominants, dominants and subdominants. As has been demonstrated by long-term studies that have been carried out in anthropogenic ponds (Strzelec et al. 2006), *P. acuta* is becoming a more and more settled species in Poland and does not affect the native fauna. Perhaps its small total density in the above-mentioned type of ponds is not a factor that influences the diversity of gastropods.

In water bodies of both the *P. acuta* and *P. antipodarum* type, an alternate dominance of leading species was demonstrated. This is confirmed by the results of the Spearman analysis. Only in water bodies of the *F. fragilis* type did we observe the presence of one alien species. In this type of pond, phytophilous species belonged to the eudominants, whereas in other types, this species often colonised both sediment-covered leaf deposits and macrophytes.

Analysis of alien species density in each type of pond proved the species’ colonisation prosperity of isolated habitats and indicated the possibility of the co-occurrence of species from different geographic regions. The densities of alien species in the woodland ponds are low in comparison to the density described by other authors (Schreiber et al. 1998; Hall et al. 2003; Kerans et al. 2005). In our study, we can specify that the occurrence of *P. antipodarum* changes the community structure of freshwater gastropods in isolated ponds in which this species is eudominant. In water bodies of this type, 11 of the 16 reported species belonged to the recedents. This is also expressed in the values of the Simpson diversity index. Further research may indicate whether this has an impact on native biodiversity. The prediction of any negative effects, particularly of invasive species, is a priority in research to prevent and control possible invaders (Hall et al. 2006).

During the winter, the woodland ponds that were studied are covered with ice, which means that the deposits of allo- and autochthonous detritus covering the bottom sediments become the habitat for gastropods to survive in, even for species that come from distant climate zones (Oertli 1995; Strzelec and Lewin 1996). Fluctuation in the water level is not a significant factor that limits the occurrence of alien species. *P. antipodarum* can bury themselves in the sediments in order to survive both dry and cold periods (Duft et al. 2003). *Ferrissia* juveniles that have not been bred yet form a septum in response to dry conditions, while *P. acuta* burrows into bottom sediments (Richardot 1974; Costil et al. 2001).

As was demonstrated in this study, only a few native species formed permanent populations in woodland ponds. *R. balthica*, which was eudominant in water bodies of the *P. antipodarum* type, is resistant to desiccation (Gérard 2001) and high water temperatures (Hubendick 1947). Thomas and Daldorph (1994) relate their high density to an increasing nutrient content that promotes primary production. The increase of trophy results in the reconstruction of algae communities into those that are characteristic to eutrophic waters.

The occurrence of alien species in isolated ponds indicates that they are effective colonisers of habitats that are a product of human activity and that remain under its influence (disturbed ecosystems). However, we cannot define the degree of these disturbances because nowadays most ecosystems on Earth are modified by human activity, and according to Loson and MacIsaac (1997), even the penetration of alien species into the environment is a source of disruption. We can assume that the alien species that were recorded in woodland ponds are in the process of expanding their range or forming a permanent population. The effects of their impact on native fauna are visible only for *P. antipodarum*, and according to Simon and Townsend (2003), they can range from undetectable to dramatic. The fact is that degraded water bodies are susceptible to invasions. They are interesting research targets because of their spatial isolation and the fact that they actively influence the diversity of the flora and fauna in industrial areas with no natural water bodies.
